# Glycogen Synthase Kinase-3 Regulates Production of Amyloid-**β** Peptides and Tau Phosphorylation in Diabetic Rat Brain

**DOI:** 10.1155/2014/878123

**Published:** 2014-04-03

**Authors:** Zhong-Sen Qu, Liang Li, Xiao-Jiang Sun, Yu-Wu Zhao, Jin Zhang, Zhi Geng, Jian-Liang Fu, Qing-Guo Ren

**Affiliations:** ^1^Department of Neurology, The Sixth People's Hospital Affiliated Shanghai Jiao Tong University, Yi Shan Road 600, Shanghai 200233, China; ^2^Department of Neurology, The Central Hospital Affiliated Qingdao University, Qingdao 266042, China; ^3^Department of Pathophysiology, Key Laboratory of Neurological Diseases of Hubei Province, Tongji Medical College, Huazhong University of Science and Technology, Wuhan 430030, China

## Abstract

The pathogenesis of diabetic neurological complications is not fully understood. Diabetes mellitus (DM) and Alzheimer's disease (AD) are characterized by amyloid deposits. Glycogen synthase kinase-3 (GSK-3) plays an important role in the pathogenesis of AD and DM. Here we tried to investigate the production of amyloid-**β** peptides (A**β**) and phosphorylation of microtubule-associated protein tau in DM rats and elucidate the role of GSK-3 and Akt (protein kinase B, PKB) in these processes. Streptozotocin injection-induced DM rats displayed an increased GSK-3 activity, decreased activity and expression of Akt. And A**β**40 and A**β**42 were found overproduced and the microtubule-associated protein tau was hyperphosphorylated in the hippocampus. Furthermore, selective inhibition of GSK-3 by lithium could attenuate the conditions of A**β** overproduction and tau hyperphosphorylation. Taken together, our studies suggest that GSK-3 regulates both the production of A**β** and the phosphorylation of tau in rat brain and may therefore contribute to DM caused AD-like neurological defects.

## 1. Introduction

Alzheimer's disease (AD) is characterized by the presence of two pathological protein deposits: extracellular senile plaques (SP) and intracellular neurofibrillary tangles (NFTs). The former is composed of *β*-amyloid (A*β*) [1] and the latter is formed by bundles of paired helical filaments (PHFs), which are mainly constituted by abnormal hyperphosphorylated tau protein [2]. Several protein kinases may take part in these pathological processes, including cyclin-dependent kinase 5 (cdk-5), GSK-3, and protein kinase A (PKA). Activated GSK-3 increases the production of A*β* peptides by promoting of *γ*-secretase activity and induces the aggregation and deposition of A*β* [3]. On the other hand, GSK-3 regulates tau hyperphosphorylation at ser198/ser199/ser202 sites and ser396/ser404 sites [4]. Furthermore, as a downregulator of insulin signaling, GSK-3 is regulated by Akt [5].

The available epidemiological data are largely inconclusive with regard to the contribution of diabetes mellitus to cognitive impairment and AD-type neurodegeneration [6, 7]. Both diabetes and AD are characterized by localized amyloid deposits that progress during the course of the diseases [8]. In hippocampal neurons of diabetic mice, the stains of A*β*40 and A*β*42 are increased, but the expression of GSK-3 is weakened by immunohistochemistry [9]. In AD-like Tg2576 mice, diet-induced insulin resistance promotes A*β*40 and A*β*42 generation in the brain. Further study suggest that PI3-kinase/PS473-Akt/PKB is reduced in these brains and suggest that GSK-3 as a downsteam kinase of these kinases may affect the production of A*β* [10]. In the skeletal muscle of type II diabetes mellitus patients, GSK-3 activity and its expression level are significantly higher [11]. Increased GSK-3 activity is also found in the development of insulin resistance and type II diabetes fat tissue of C57BL/6J mice [12]. Immunohistochemical results show that ser198/ser199/ser202 sites are hyperphosphorylated in the hippocampus of diabetic mice, but the expression of GSK-3 is reduced [13]. In the brain of insulin knockout mice, hyperphosphorylation of tau at threonine 231 and of the neurofilament is exhibited, but GSK-3*β* activity was inhibited [14].

All of the above evidences imply that GSK-3 may be linked to both A*β* production and tau hyperphosphorylation during the course of diabetes. To better understand the mechanisms of AD-like changes in diabetic rat brain, we investigated the activity of GSK-3 and Akt and the expression of Akt; then A*β* production and tau phosphorylation was determined. Furthermore, the role of GSK-3 was explored by using LiCl as a specific inhibitor [15].

## 2. Materials and Methods

### 2.1. Antibodies and Chemicals

The primary A*β* antibodies G2-10 (capture antibody for A*β*_40_), G2-11 (capture antibody for A*β*_42_), and WO_2_ (detection antibody) used for ELISA were purchased from A*β*eta Company (Germany), and standard synthetic A*β* peptides were obtained from Sigma (St. Louis, MO, USA). Antibodies against phospho-Ser^473^ Akt and total Akt were purchased from Cell Signaling technology (Beverly, MA, USA). Monoclonal antibodies PHF-1, which labels tau phosphorylated at Ser-396 and/or Ser-404, and Tau-1, which labels tau where neither serines 198, 199, or 202 are phosphorylated, were gifts from Dr. Peter Davies (Albert Einstein College of Medicine, Bronx, NY, USA) and Dr. Lester Binder (Northwestern University, Chicago, IL, USA), respectively. Polyclonal antibody 111e, which reacts with the total tau, was a kind gift from Drs. Iqbal K and Grundke-Iqbal I (New York State Institute for Basic Research, Staten Island, NY, USA). Phospho-GS peptide, a specific GSK-3 substrate, was obtained from Upstate Biotechnology Inc. (Lake Placid, NY, USA). Bicinchoninic acid (BCA) protein detection kit and TMB were obtained from Pierce (Rockford, IL, USA). [*γ*-^32^P] ATP was obtained from Beijing Yahui Biologic and Medicinal Engineering Co. (Beijing, China). Goat anti-rabbit or goat anti-mouse peroxidase-conjugated secondary antibodies and phosphocellulose paper were obtained from Pierce Chemical Co. (Rockford, IL, USA). Polyclonal and monoclonal Histostain-SP kits were obtained from Zymed Laboratories Inc. (South San Francisco, CA, USA). Streptozotocin (STZ), diaminobenzidine, LiCl (a specific inhibitor of GSK-3), and other chemicals were purchased from Sigma (St. Louis, MO, USA).

### 2.2. Establishment of DM Rat Model

Four-month-old Sprague-Dawley rats (Grade II, male, weight 200–250 g, supplied by Experimental Animal Center of Tongji Medical College) were divided into 4 groups: control group, diabetes mellitus (DM) group, DM plus NaCl group, and DM plus LiCl group by random. Except for control rats, the other rats received intraperitoneal injection with 55 mg/kg streptozotocin (STZ). The DM rats were determined by fasting blood glucose ≥16.7 mmol/72 h after streptozotocin injection. DM plus NaCl group: 24 h following STZ injection, the rats were administered by intraperitoneal injection 0.4 mL of 0.3 M NaCl for 10 days. DM plus LiCl group: 24 h following STZ injection, the rats were injected intraperitoneally with 400 *μ*L of 0.3 M LiCl for 10 days.

### 2.3. Preparation of Rat Hippocampal Extracts

Following continuous injection of NaCl or LiCl for 10 days, the rats were killed. The hippocampus was immediately removed and homogenized at 4°C using a Teflon glass homogenizer in 50 mmol Tris-HCl, pH 7.4, 150 mmol NaCl, 10 mmol NaF, 1 mmol Na_3_VO_4_, 10 mmol *β*-mercaptoethanol, 5 mmol EDTA, 2 mmol benzamidine, 1.0 mmol phenylmethylsulfonyl fluoride, 5 *μ*g/mL leupeptin, 5 *μ*g/mL aprotinin, and 2 *μ*g/mL pepstatin. The tissue homogenates were then divided into two portions. One portion of each homogenate was centrifuged at 12,000 ×g for 20 min at 4°C, and the resulting supernatant was stored at −80°C for assaying activities of protein kinases. The other portion was mixed in 2 : 1 (v/v) ratio with lysis buffer containing 200 mmol Tris-HCl, pH 7.6, 8% SDS, 40% glycerol, boiled for 10 min in a water bath, and then centrifuged at 12,000 ×g for 30 min, and the supernatant was stored at −80°C for Western blot analysis. The concentration of protein in the hippocampal extracts was measured by BCA kit according to the manufacturer's instructions (Pierce, Cheshire, UK).

### 2.4. Assay of GSK-3 and Akt Activity

The GSK-3 activity in rat hippocampal exacts was measured using phospho-GS peptide 2 (Upstate, Lake Placid, NY, USA) as described previously [16]. Briefly, tissue extracts, 7.5 *μ*g proteins were incubated for 30 min at 30°C with 20 *μ*M peptide substrate and 200 *μ*mol [*γ*-^32^P] ATP (1,500 cpm/pmol ATP) in 30 mmol Tris, pH 7.4, 10 mmol MgCl_2_, 10 mmol NaF, 1 mmol Na_3_VO_4_, 2 mmol EGTA, and 10 mmol *β*-mercaptoethanol in a total volume of 25 *μ*L. The reaction was stopped by addition of 25 *μ*L of 300 mM *O*-phosphoric acid. The reaction mixture was applied in triplicates on phosphocellulose paper (pierce). The filters were washed three times with 75 mmol *O*-phosphoric acid, dried, and counted by liquid scintillation counter. GSK-3 activities was calculated with picomoles of phosphate incorporated/mg protein/min at 30°C and expressed as relative activity against control. The Akt activity was measured using histone 2B as a substrate as described previously [17, 18]. Briefly, after the immunoprecipitates were washed with lysis buffer and kinase buffer, 40 *μ*L kinase buffer containing 200 *μ*M [*γ*-32P] (5 *μ*Ci), 100 *μ*M ATP, 1 *μ*g/*μ*L histone 2B; then the samples were incubated at 30°C for 15 min and spotted onto P81 filter papers; the filter papers were washed by 75 mM *O*-phosphoric acid, dried, and counted by liquid scintillation counter. Akt activity was also expressed as relative activity against control.

### 2.5. Measurement of A*β* in Hippocampus of the Rats

The A*β*40 and A*β*42 in the hippocampus were measured by a Sandwich enzyme-linked immunosorbent assay (ELISA) using antibodies as described previously [19]. Experiments were performed in a 96-well plate. Briefly, affinity-purified mAb G2-10 (0.5 *μ*g/well) was applied as the capture antibody for A*β*_40_, mAb G2-11 (1 *μ*g/well) was used as a capture antibody for A*β*_42_, and mAb WO_2_ was used as a detection antibody. Neutravidin-horseradish peroxidase and TMB were used for reporter system and absorbance values at 450 nm were determined with microplate reader (TECAN, Austria). Levels of A*β* were expressed as a relative level against control. Each sample was tested in triplicate in each experiment.

### 2.6. Evaluation of Tau Phosphorylation and Expression of Akt

The phosphorylation of tau at various sites was determined by Western blot as described formerly [20]. For immunoblotting, about 20 *μ*g of proteins were loaded in each lane. Proteins were separated by SDS-PAGE and transferred to polyvinylidene difluoride membranes (Amersham Pharmacia Biotech, NJ, USA). After being blocked for 1 h in a solution of 5% nonfat dry milk in TBS/Tween 20, membranes were immunoblotted using primary antibodies PHF-1 (1 : 500), Tau-1 (1 : 30,000), and 111e (1 : 3,000) at 4°C overnight and developed with alkaline phosphatase-labeled IgG (0.5 g/mL, Amersham Pharmacia, NJ, USA) as secondary antibodies. 5-bromo-4-chloro-3-indolyl-phosphate/nitro blue tetrazolium (BCIP/NBT) was used as substrate [2]. Detection of the phosphorylation of Akt was performed with antibodies to phospho-Ser^473^ Akt and total Akt (Cell Signaling technology) (dilution 1 : 800); immunoblots were developed using horse radish peroxidase-conjugated goat anti-rabbit IgG (1 : 2000) followed by detection with enhanced chemiluminescence.

### 2.7. Statistical Analysis

The intensity of the protein bands from Western blot was analyzed by Image-Pro Plus software and ID Image Analysis software (KODAK, USA), respectively. The data were presented as mean ± SD and were analyzed by analysis of variance.

## 3. Results

### 3.1. Measurement the Level of Serum Glucose in Rats

**Figure 1:**
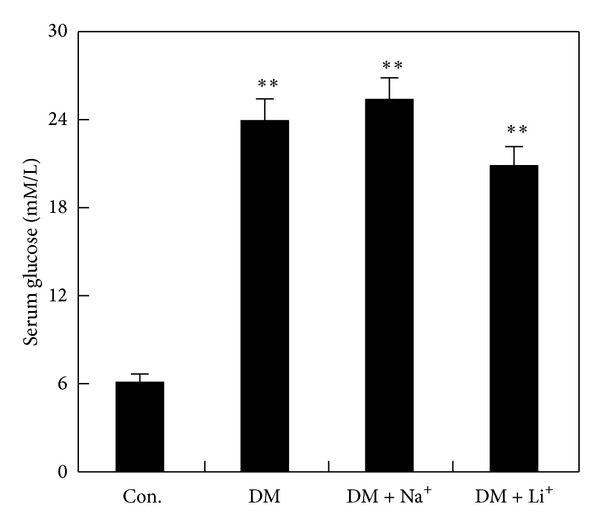
The level of serum glucose of the rats. 72 h following streptozotocin injection, the levels of serum glucose in DM group, DM plus NaCl group, and DM plus LiCl group increased obviously. **P < 0.01 versus control group.

72 h after STZ injection, the fasting blood glucose (FBG) of the rats among DM group, DM plus NaCl group, and DM plus LiCl group was over 16.7 mmol/L, and it was increased obviously as compared with controls. The fasting blood glucose of the rats in DM plus LiCl group was not induced a significant decrease when compared with DM group, but there was a tendency toward decrease (Figure 1).

### 3.2. Assay of the Activity of GSK-3 and Akt

**Figure 2:**
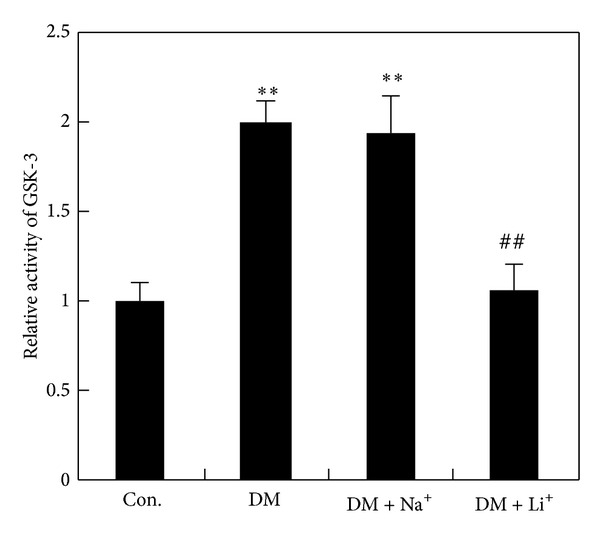
The activity of GSK-3. The activities of GSK-3 in hippocampal extracts from rats injected with STZ, STZ plus NaCl, or STZ plus LiCl were determined by using specific peptide substrates. **P < 0.01 versus control group, ^##^ *P* < 0.01 versus DM group.

**Figure 3:**
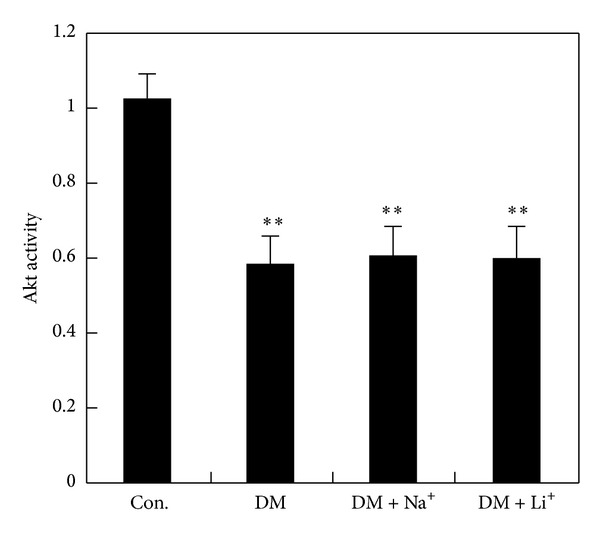
The activity of Akt. The activities of Akt in hippocampal extracts from rats injected with STZ, STZ plus NaCl, or STZ plus LiCl were determined by using specific peptide substrates. **P < 0.01 versus control group.

To reveal the role of GSK-3 and Akt during the course of diabetes, activity of GSK-3 and Akt was measured (Figure 3). GSK-3 is an important kinase in the process of A*β* production and tau hyperphosphorylation [3, 4]. GSK-3 was inactivated by phosphorylation of serine 9 in GSK-3*β* and serine 21 in GSK-3*α* [18]; Akt appears to be the predominant kinase mediating this phosphorylation of GSK-3. Both brain GSK-3 and Akt can be regulated by blood glucose in mice [18]. In our study, we found the strong activity of GSK-3 and lower activity in rat hippocampus when fasting blood glucose was highly increased by STZ intraperitoneal injection. After treating the DM rats with LiCl, GSK-3 activity was decreased 46% approximately. No apparent inhibition of GSK-3 activity was observed after treating the DM rats with NaCl (Figure 2). There were not obvious changes of Akt activity after treatment of LiCl or NaCl with DM rats. These results implied that hyperglycemia induced strong activity of GSK-3 and lower activity of Akt in rat brain. LiCl could directly inhibit the activity of GSK-3 rather than Akt.

### 3.3. Assay Production of A*β* and Tau Phosphorylation and the Role of LiCl

**Figure 4:**
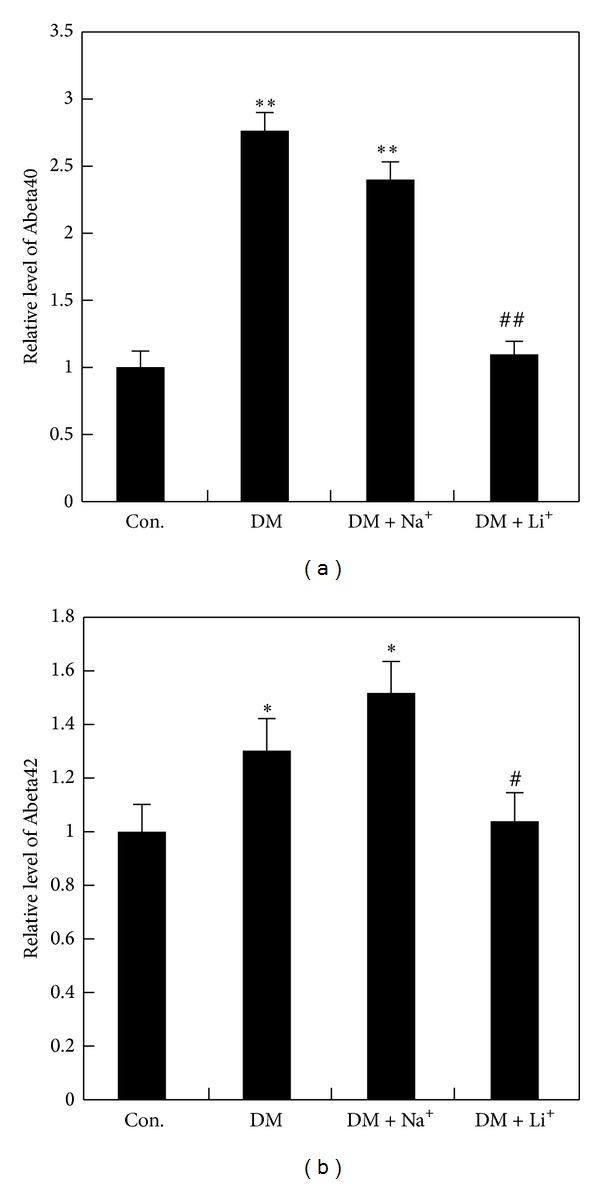
Production of A*β*40 (a) and A*β*42 (b) generation. STZ treatment increased A*β*40 and A*β*42 production significantly and LiCl could reverse the change induced by STZ treatment. *P < 0.05 and **P < 0.01 versus control group, ^#^ *P* < 0.05 and ^##^ *P* < 0.01 versus DM group.

A*β* deposition is an important mechanism in both AD and diabetes, and GSK-3 plays an important role in regulation of A*β* production. The high level of GSK-3 activity and low level of Akt activity were found in our study. To elucidate the effects of GSK-3 and Akt on A*β* production, A*β* production was determined following measurement of GSK-3 and Akt activity. As shown in Figure 4, the production of A*β*40 (Figure 4(a)) and A*β*42 (Figure 4(b)) was increased significantly while activation of GSK-3 and inhibition of Akt were induced in DM group. After treating the DM rats with LiCl, the production of A*β*40 and A*β* 42 was reduced by 60% and 21%, respectively. There was not significant reduction of A*β*40 and A*β*42 following NaCl treatment. These data showed that production of A*β*40 and A*β*42 is increased in the hippocampus of DM rats; both activation of GSK-3 and inhibition of Akt might play an important role in this process.

**Figure 5:**
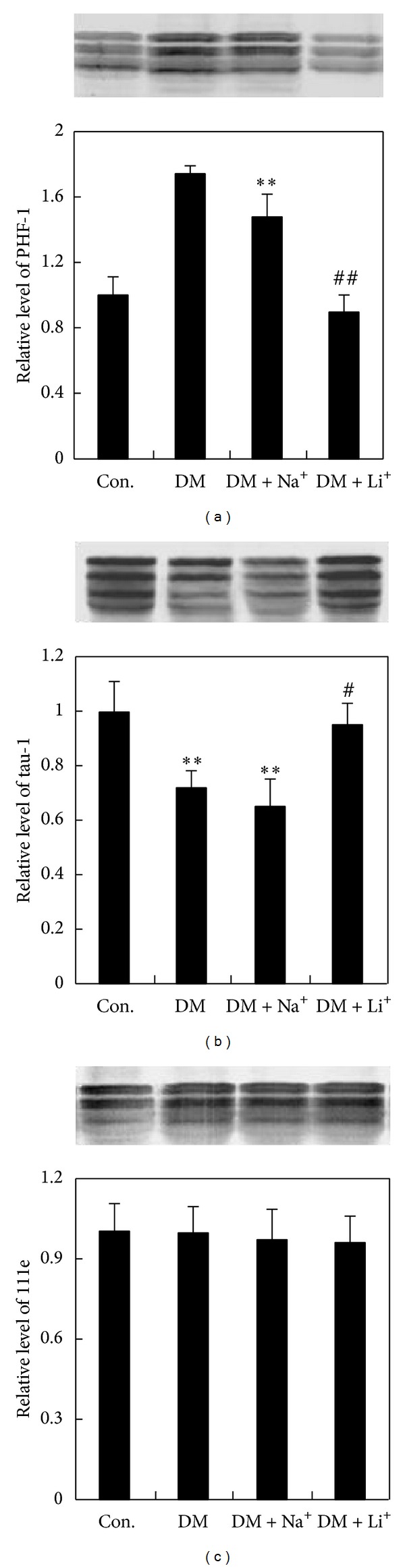
Tau phosphorylation. *P < 0.05 and **P < 0.01 versus control group, ^#^ *P* < 0.05 and ^##^ *P* < 0.01 versus DM group.

GSK-3 is also an important kinase in the regulation of tau phosphorylation. When itwas activated, tau was prone to phosphorylation at ser198/ser199/ser202 sites and ser396/ser404 sites. Hence, we examined the state of tau phosphorylation in DM rat by Western blot using phosphorylation-dependent and site-specific tau antibodies. We found that in DM rat hippocampus, the immunoreactivity of PHF-1 (detection of ser396/ser404, phosphorylated sites) was increased obviously as compared with the control group, but the staining of PHF-1 was reversed after treatment of LiCl (Figure 5(a)). Moreover, the immunoreactivity of Tau-1 (detection of ser198/ser199/ser202, nonphosphorylated sites) was decreased in DM rat as compared with controls, and LiCl could reverse this staining (Figure 5(b)). NaCl treatment did not change the staining of PHF-1 and Tau-1 in the DM group. The total level of tau measured by R111e was not changed significantly in all the four groups (Figure 5(c)).

### 3.4. Assay of Akt Expression

**Figure 6:**
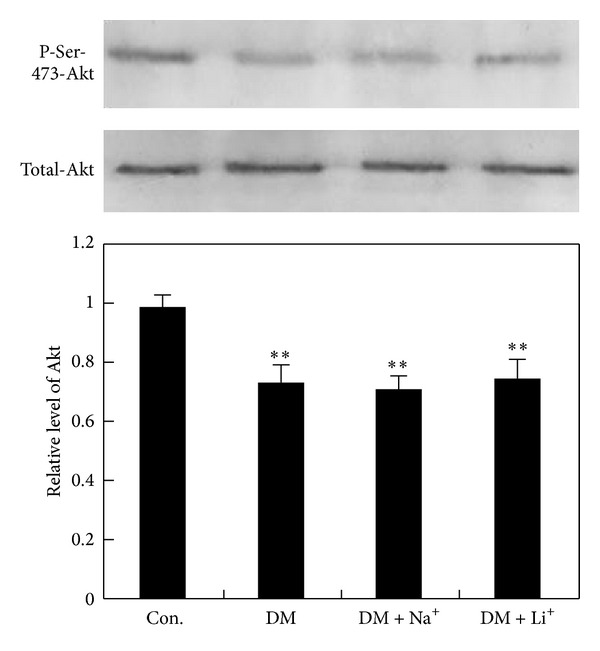
Akt expression. **P < 0.01 versus control group.

GSK-3 was inactivated by Akt by phosphorylation at serine 9 and serine 21. To test if Akt was regulated by streptozotocin-induced hyperglycemia and was regulated by activation of GSK-3, we examined phosphorylation level of Akt. Our results show that the phosphorylation of Akt at Ser473 site was deceased in DM rat hippocampus, and both LiCl and NaCl administration could not recover the decrease, and the total level of Akt was not changed. These results suggest that GSK-3 was inhibited by LiCl instead of Akt inhibition (Figure 6).

### 3.5. Behavioral Testing

**Figure 7:**
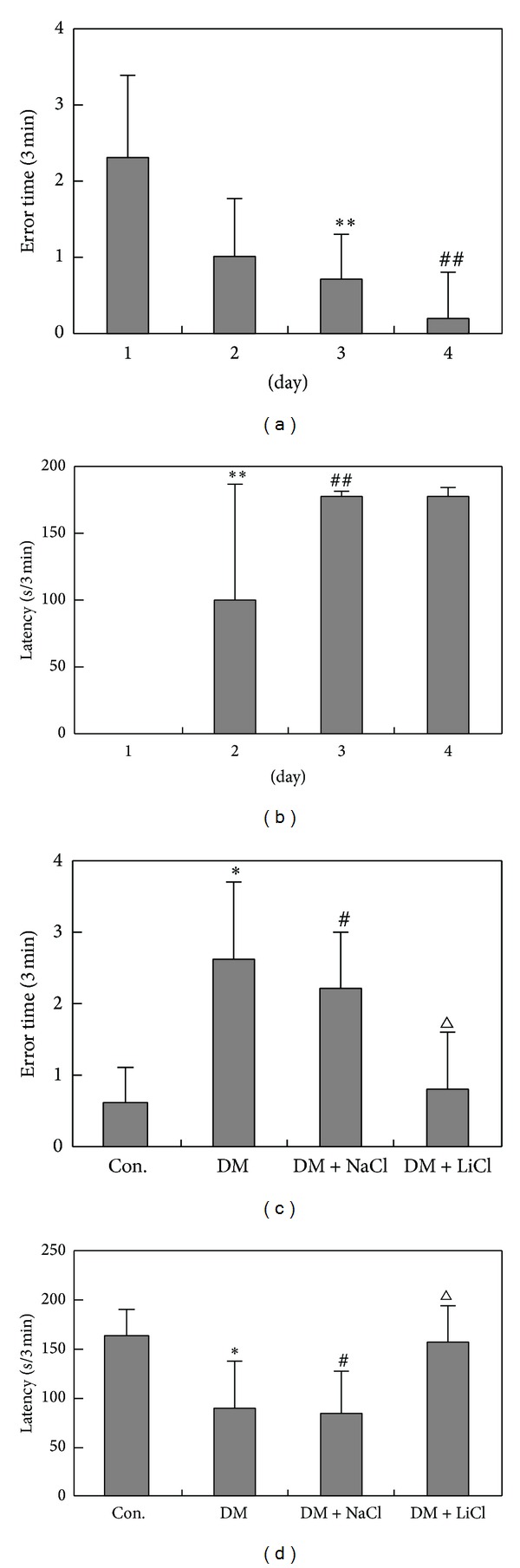
Step-down electronic inhibitory avoidance task. *P < 0.05, ^#^ *P* < 0.05 versus Con. group, ^△^ *P* < 0.05 versus DM group. (a) The error time before modeling. The error time reached the similar time level after the rats were trained for 4 d. **P < 0.01, ^##^ *P* < 0.01 versus 1 d. (b) The latency before modeling. The latency was increased after the rats were trained for 3 d and 4 d. **P < 0.01, ^##^ *P* < 0.01 versus 4 d. (c) The error time at 10 days after LiCl administration. The error time increased in DM group but decreased in group with LiCl administration for 10 days. *P < 0.05, ^#^ *P* < 0.05 versus Con. group, ^△^ *P* < 0.05 versus DM group. (d) The latency at 10 days after LiCl administration. The latency decreased in DM group but increased in group treated with LiCl administration for 10 days. *P < 0.05, ^#^ *P* < 0.05 versus Con, ^△^ *P* < 0.05 versus DM.

To further explore the cognition dysfunction caused by the changes of Akt and GSK-3 and Tau hyperphosphorylation in DM rats, the step-down electronic inhibitory avoidance task was assessed in rats. To examine which rats have lower ability of learning and memory, we first trained all rats to stay on the platform for 3 min and not to step down. Ninety-four hours after the training, latencies to step-down during training session were not significantly different across the groups (data are not shown because the latency to step-down in this session was basically nonexistent). The results suggest significantly shorter latencies and more error times to step-down in DM rat when compared to control groups (*P* < 0.05). Longer latencies and less error times were observed in LiCl administration to DM rat as compared with DM rat (*P* < 0.05). There is no significant difference between NaCl administration to DM rats and DM rats (*P* > 0.05). These results suggest that inhibition of LiCl on GSK-3 might improve the memory of DM rat (Figure 7).

## 4. Discussion

Study and memory dysfunction are the main phenomena of central nervous system complications in type I diabetes mellitus [21]. Cerebral atrophy, which is characterized in AD patients, is also found in young patients with type 1 diabetes who are otherwise healthy [22]. In experimental animal models, an increase of stains with A*β*40 and A*β*42 antibody is induced in DM mice hippocampus [9]. Tau is also hyperphosphorylated at ser199/ser202 while the expression of GSK-3 is decreased in DM mice hippocampus [13]. Whether A*β* overproduction and tau hyperphosphorylation happen synchronously in the hippocampus of DM rat is puzzling, and the role of GSK-3 and Akt in these processes is not clear.

Both GSK-3 and Akt in mice brain could be regulated by alterations of blood glucose; streptozotocin-induced hyperglycemia brain increases Akt activity and decreases GSK-3 activity, which could be reversed by lowering blood glucose with insulin administration [18]. In an experimental model related to sporadic Alzheimer's disease, after intracerebroventricular injection of streptozotocin for 1 month, there is a decrease of GSK-3 alpha/beta activity in the rat hippocampus [5]. In our study, the hyperglycemia was induced by intraperitoneal injection of rats with streptozotocin, but an increase of GSK-3 activity and a decrease of Akt activity were induced in the rat hippocampus. Hence, we proposed that hyperglycemia might affect the activities of GSK-3 and Akt in DM rat brain. In AD-like Tg2576 mice, diet-induced insulin resistance promotes Abeta40 and Abeta42 peptide generation in the brain that corresponds with increased gamma-secretase activities. Further exploration of the apparent interrelationship of insulin resistance to brain amyloidosis reveals a functional decrease in insulin receptor- (IR-) mediated signal transduction in the brain; Akt/PKB inhibits glycogen synthase kinase (GSK-3 alpha) activity [3]. We also found that there was an increase of A*β* production when GSK-3 activity was increased and PKB activity was decreased in the hippocampus of DM rats. Lithium, a specific inhibitor of GSK-3*α* and GSK-3*β*, reduces A*β* production by interfering with APP cleavage at the *γ*-secretase step and is found to reduce A*β* production in the mice expressing pathogenic familial Alzheimer's disease [3]. In our study, after treating DM rats with LiCl, GSK-3 activity was decreased significantly in the hippocampus, and A*β*40 and A*β*42 were reduced by 60% and 21%, but administration of DM rats with NaCl reduced neither the activation of GSK-3 nor the overproduction of A*β*40 and A*β*42. The low level activity of Akt maintained in LiCl administration showed that lithium could not inhibit Akt in DM rat brain. These data suggested that GSK-3 played an important role in A*β* overproduction of DM rat hippocampus.

On the other hand, LiCl, as a specific GSK-3 inhibitor, has been confirmed to block tau hyperphosphorylation either in culture neuron or in rat brain [23, 24]. The major kinase for tau phosphorylation is GSK3*β*. Smaller contributions of GSK3*α*, cdk-5, and MAPK are suggested [25]. In DM mice hippocampus, tau is hyperphosphorylated at ser199/ser202 sites with lower GSK-3 expression [13]_._ In our study, we found that tau hyperphosphorylation was induced at ser198/ser199/ser202, Ser396/Ser404 sites just when the activity of GSK-3 increased highly. When the DM rats were treated with LiCl, GSK-3 activity decreased about 46%, and hyperphosphorylation tau was reversed at ser396/ser404, ser198/ser199/ser202 sites in the hippocampus. These sites are just the targets of GSK-3 [26], while NaCl treatment showed no apparent changes in these sites as compared with DM rats. These data also suggested that LiCl reduced tau hyperphosphorylation at ser396/ser404, ser198/ser199/ser202 in DM rat hippocampus by inhibition of GSK-3 rather than of Akt (PKB).

Tau hyperphosphorylation may be associated with cognitive impairment [27, 28]. Whether the changes of Akt and GSK-3 may reduce memory retention in DM rat is not sure. Our results suggest that the increase of GSK-3 may be response for the impairment of step-down inhibitory avoidance task rather than Akt because the activities and the expression of Akt were not significantly different among DM group, DM + NaCl group, and DM + LiCl group, but other studies show that learning impairment and hippocampal ERK and Akt inactivation are induced by scopolamine in male Sprague-Dawley rats [29].

In conclusion, our results demonstrate that GSK-3 has an important role in the pathogenesis of diabetic neurological complications byregulation of A*β* production and tau hyperphosphorylation, and the present data suggest that GSK-3 might be a key target in the therapy of central nervous system neuropathy of diabetes mellitus.
